# Exploring Dystrophin Expression and Mutations in the *DMD* and Dystrophin-Glycoprotein Complex Genes as Prognostic Factors in Leiomyosarcomas

**DOI:** 10.3390/ijms27125290

**Published:** 2026-06-11

**Authors:** Juliana Salazar, Paula Cerdà, Alícia Artigas, Ruth Orellana, Allan González, Raúl Terés, María J. Arranz, Silvia Bagué, Caterina Fumagalli, José Antonio González, Ana Peiró, Lidia González-Quereda, María José Rodríguez, Eduard Gallardo, María Aguado, Ana Sebio

**Affiliations:** 1 Translational Medical Oncology Laboratory, Institut de Recerca Sant Pau (IR Sant Pau), 08041 Barcelona, Spain; pcerda@santpau.cat (P.C.); rteres@santpau.cat (R.T.); maguados@santpau.cat (M.A.); 2 Department of Medical Oncology, Hospital de la Santa Creu i Sant Pau, 08041 Barcelona, Spain; 3 Institut de Recerca Sant Pau (IR Sant Pau), 08041 Barcelona, Spain; 4 Department of Pathology, Hospital de la Santa Creu i Sant Pau, 08041 Barcelona, Spain; 5 Research Laboratory Unit, Fundació Docència i Recerca Mútua Terrassa, 08221 Terrassa, Spain; mjarranz@mutuaterrassa.es; 6 General Surgery Unit, Hospital de la Santa Creu i Sant Pau, 08041 Barcelona, Spain; 7 Department of Orthopedic and Traumatology Surgery, Hospital de la Santa Creu i Sant Pau, 08041 Barcelona, Spain; 8 Department of Genetics, Hospital de la Santa Creu i Sant Pau, 08041 Barcelona, Spain; 9 U762 CIBERER (Rare Diseases Network Research Centre), Instituto de Salud Carlos III, 28029 Madrid, Spain; 10 Laboratori de Malalties Neuromusculars, Institut de Recerca Sant Pau (IR Sant Pau), 08041 Barcelona, Spain

**Keywords:** leiomyosarcomas, prognosis, biomarker, dystrophin, dystrophin–glycoprotein complex

## Abstract

Molecular factors influencing prognosis in leiomyosarcomas (LMS) remain poorly understood. Given that LMS arise from smooth muscle and that the dystrophin gene (*DMD*) has been suggested as a tumor suppressor in this tumor type, this study investigated dystrophin expression and somatic mutations in *DMD*, and dystrophin–glycoprotein complex (DGC) and dysferlin (*DYSF*) coding genes as prognostic biomarkers in LMS. Seventy-seven patients with LMS were retrospectively included in the study. Dystrophin expression was evaluated by immunohistochemistry. The presence of *DMD* point mutations were determined using next-generation sequencing (NGS), and *DMD* copy-number variants were assessed by multiplex ligation-dependent probe amplification (MLPA) technique. Low dystrophin expression was observed in 66% of tumors and showed a nominal association with tumor grade, with 76% of grade 3, 58% of grade 2, and 33% of grade 1 tumors showing low dystrophin expression (*p* = 0.046). Dystrophin expression was not associated with overall survival (OS), nor with recurrence-free survival (RFS) in localized cases or progression-free survival (PFS) in metastatic cases. However, dystrophin expression was significantly associated with shorter OS (*p* = 0.006) and RFS (*p* = 0.003) in patients with localized grade 2 tumors. Mutations in *DMD*, DGC-coding genes and *DYSF* were identified in 59% of tumors, but no significant associations were found with pathological, molecular, or survival data. Loss of dystrophin expression was common in LMS tumors and was associated with high-grade tumors in our cohort. Nonetheless, the role of dystrophin as a prognostic biomarker of survival was only observed in patients with localized grade 2 tumors in this exploratory, hypothesis-generating study, and therefore needs further validation.

## 1. Introduction

Leiomyosarcomas (LMS) are rare tumors arising from smooth muscle tissue and account for around 20% of soft tissue sarcomas (STS). The most common sites for LMS to develop are the extremities, uterus and retroperitoneum. LMS are aggressive tumors with a poor prognosis, and up to 40% of the patients will die regardless of treatment [1,2]. Known prognostic factors are limited to clinical criteria including location, stage, size, tumor depth, histological grade, the number of metastatic lesions and the interval between primary tumor treatment and metastasis appearance [3]. The treatment of localized LMS is wide margin surgery. Perioperative radiotherapy reduces local recurrence without affecting survival [4]. Perioperative chemotherapy has shown a significant survival benefit in high-risk localized LMS of the extremities and trunk wall [5,6], but there is a lack of evidence for other sites like the retroperitoneum and uterus. In the metastatic disease, doxorubicin monotherapy and the new combination with trabectedin are currently the preferred first-line treatment [7].

Somatic molecular alterations such as whole-genome duplication and copy-number variants (CNVs) are common in LMS, along with alterations in tumor suppressor genes like *RB1*, *PTEN*, and *TP53*, and in genes involved in chromatin remodeling such as *ATRX*, and in genes participating in homologous recombination [8]. Despite the characterization of these genomic alterations, no specific genomic or molecular markers to aid diagnosis, prognosis, or treatment have been identified in LMS, contrary to other sarcomas [9]. Interestingly, the dystrophin gene (*DMD*) has been proposed as a suppressor gene in myogenic and non-myogenic tumors [10,11]. Dystrophin, in association with proteins of the dystrophin–glycoprotein complex (DGC), connects the cytoskeleton to the extracellular matrix at the sarcolemma [12,13] and mediates signal transduction pathways involved in various cellular processes, including cell survival [14]. A study in STS of myogenic origin showed that *DMD* deletions were associated with progression to high-grade lethal sarcomas, and that dystrophin expression was low or undetectable in 62% of LMS cases [10]. *DMD* deletions and/or loss of dystrophin expression were also correlated with metastatic progression [15] and worse clinical outcomes [16,17] in LMS. In contrast, leiomyomas, which are benign tumors, showed no deletions in *DMD* or loss of dystrophin expression [10].

Based on this knowledge and the limited evidence available in LMS, this study aimed to investigate dystrophin expression and mutations in *DMD*, related DGC-coding genes, and dysferlin (*DYSF*), as potential biomarkers of tumor aggressiveness and patient survival in a well-characterized cohort of patients with localized or metastatic LMS.

## 2. Results

### 2.1. Patient Characteristics and Clinical Variant Assessment

A total of 77 patients with LMS were included for analyses (Table 1). Tumor sites were extremities (40%), uterus (33%), and retroperitoneum (27%). At inclusion, 64% of participants had localized disease, and the remaining patients had metastatic disease. The median follow-up was 45.2 months (interquartile range, 18.7–91.9). The median overall survival (OS) was 47 months [95% confidence interval (CI) 35.2–58.7]. Patients with localized disease had a median OS of 109.1 months (95% CI 59.7–158.4) versus 33.3 months (95% CI 12.4–54.3) for patients with metastatic disease at diagnosis (*p* < 0.001). The median recurrence-free survival (RFS) of patients with localized disease was 33.3 months (95% CI 21.7–44.8), and the median progression-free survival (PFS) of patients with metastatic disease was 12.8 months (95% CI 8.8–16.7). OS for patients with grade 1 tumors was 109.1 months [95% CI not applicable (NA)–NA], 96.3 months (95% CI 37.6–155.1) for patients with grade 2, and 37.4 months (95% CI 28.8–46.1) for patients with grade 3 tumors (*p* = 0.04).

### 2.2. Dystrophin Expression in LMS Tumors

**Figure 1:**
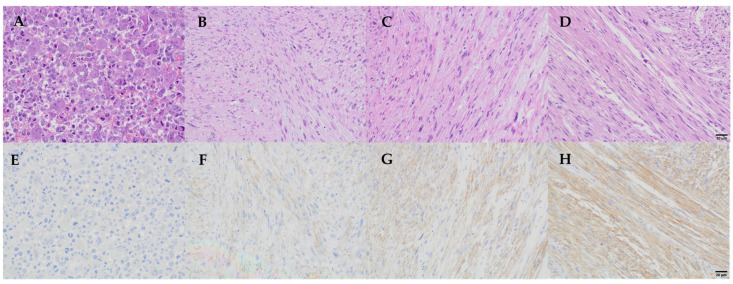
Representative images of leiomyosarcoma tissues (400×) (**A**–**D**) hematoxylin and eosin, and (**E**–**H**) immunohistochemical staining for dystrophin protein expression (NCL-DYSA; Novocastra Laboratories Ltd., Newcastle Upon Tyne, UK). The percentage of positive tumor cells was scored from 0% to 100%, and the intensity was graded as (**E**) negative (score 0), (**F**) weak (score 1), (**G**) moderate (score 2), or (**H**) strong (score 3).

Dystrophin evaluation by immunohistochemistry (IHC) was successful in 76 of 77 samples. Figure 1 depicts dystrophin IHC staining scored as negative (0), weak (1), moderate (2), or strong (3). Low (intensity score 0 to 1) dystrophin expression was observed in 51 (66%) tumor samples and high (intensity score 2 to 3) dystrophin expression in 25 (33%). The analyses showed a nominally significant association between dystrophin expression and tumor grade. Low dystrophin expression was observed in 33% (n = 1) of grade 1, in 58% (n = 18) of grade 2, and in 76% (n = 32) of grade 3 tumors (*p* = 0.046, Wald = 3.94, df = 1) (Table 2).

**Figure 2:**
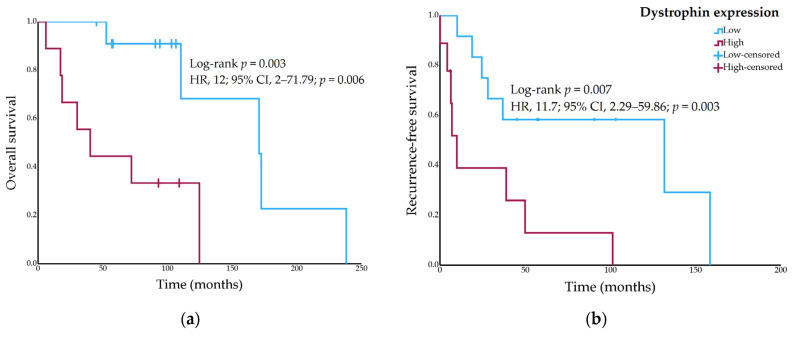
(**a**) Overall survival and (**b**) recurrence-free survival according to tumoral dystrophin expression in patients with localized grade 2 leiomyosarcoma tumors (n = 21).

Kaplan–Meier survival analyses revealed no significant association between dystrophin expression and OS in patients with localized [hazard ratio (HR), 1.32; 95% CI, 0.57–3.02; *p* = 0.52] or metastatic disease (HR, 1.84; 95% CI, 0.75–4.5; *p* = 0.18). Non-significant results were also found for RFS in localized patients (HR, 1.78; 95% CI, 0.88–3.6; *p* = 0.10) and PFS in metastatic patients (HR, 1.69; 95% CI, 0.66–4.35; *p* = 0.27). The unpredictable clinical course of grade 2 tumors, along with the linear association between dystrophin expression and histological grade prompted additional survival analyses. Patients with localized grade 2 tumors (n = 21) and high tumoral dystrophin expression had significantly shorter OS (*p* = 0.003) and RFS (*p* = 0.007) compared to those with low dystrophin expression (Figure 2). These associations remained significant after adjusting for primary tumor location in OS (HR, 12; 95% CI, 2–71.79; *p* = 0.006) and RFS (HR, 11.7; 95% CI, 2.29–59.86; *p* = 0.003). No significant associations were identified in metastatic patients.

### 2.3. Mutations and CNV in the DMD and DGC-Coding Genes in LMS Tumors

**Figure 3:**
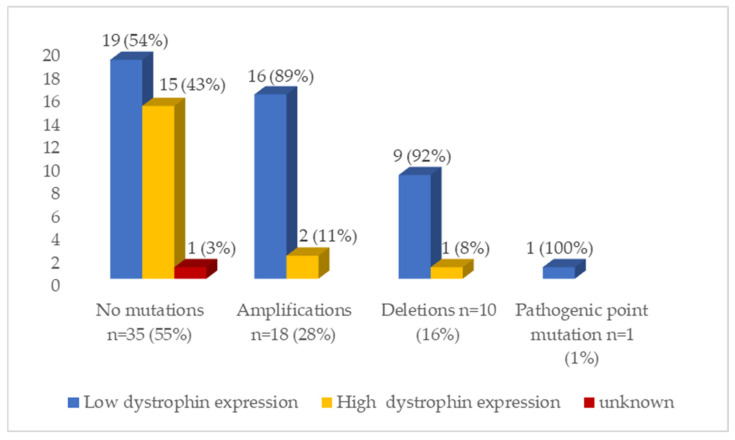
Representation of the correlation between the mutational status of DMD, including deletions, amplifications, and the stop-gained point mutation, and dystrophin expression in leiomyosarcoma tumors (n = 64).

The determination of point mutations in the 13 sequenced genes (see list of genes in point 4.6.) was performed in all samples. However, eight DNA samples did not meet the quality standards required for MLPA technique, and the CNVs of *DMD* were not determined in these samples. Five samples showed multiple altered exons (deleted and amplified exons) in *DMD* and were deemed unreliable and excluded from the analyses. Overall, CNVs in *DMD* were reported in 64 tumor samples. Ten samples harbored *DMD* deletions (16%) and 18 samples *DMD* amplifications (28%) (Table 3). The *DMD* point mutations identified are shown in Table 4. A stop-gained *DMD* mutation (p.Tyr2141Ter), which involved a codon change from TAC to TAA in exon 44, was present in a sample of extremity leiomyosarcoma. This sample had undetectable dystrophin expression, which is suggestive of pathogenicity. The *DMD* mutational status, including deletions, amplifications and the stop-gained point mutation, was correlated with low dystrophin expression (OR, 6.67; 95% CI, 1.72–25, *p* = 0.003). Figure 3 illustrates the relationship between mutations in the *DMD* gene and dystrophin expression levels in our LMS tumor cohort. Of the 64 tumor samples analyzed, 45 exhibited low dystrophin expression, and pathogenic *DMD* mutations were identified in 26 (58%) of these. Three additional missense mutations in *DMD* (p.Lys3605Asn, p.Phe590Ile and p.Leu2734Met) were also identified.

Genetic variants, including possible pathogenic or deleterious missense or 3′-UTR variants, were identified in six genes other than *DMD* (Table 4). *UTRN* was the most frequently mutated gene, found in six cases, followed by *DYSF* in five cases. Two uterine LMS samples presented possible pathogenic mutations: one sample exhibited a stop-gained mutation in *UTRN* (p.Arg191Ter), and the other a frameshift mutation in *SGCD* (p.Gly81GlufsTer32). In both tumors, these alterations occurred concomitantly with *DMD* mutations and low dystrophin expression. Twelve missense variants were also identified in the *DYSF*, *LAMA2*, *SGCG*, *SNTB1*, and *UTRN* genes, along with a 3′-UTR variant in the *DYSF* gene (c.*53G>A), which was the most common alteration (n = 3). These alterations were observed in 18 cases, including three tumors carrying missense variants that also harbored concomitant *DMD* mutations, two of which showed low dystrophin expression (Table 4).

Association analyses with clinical and pathological variables were performed to assess whether carrying mutations in *DMD* and DGC-coding genes could help identify patients at higher risk of poor prognosis. The assessment of the mutational status included CNVs in the *DMD* (Table 3), pathogenic point mutations, and possible deleterious missense and 3′-UTR variants in *DMD* and DGC-coding genes (Table 4). Thirty-eight of the 64 patients (59%) had tumors carrying at least one mutation in these genes. No significant associations were found with primary tumor location, stage, or tumor grade (Table 2). However, a significant association was found between mutational status and sex, with 70% of females with mutated tumors, compared to 33% of males (OR, 4.57; 95% CI, 1.43–14.71; *p* = 0.008). Survival analyses showed no significant association between mutational status and OS for localized (HR, 0.67; 95% CI, 0.30–1.51; *p* = 0.33) or metastatic (HR, 1.56; 95% CI, 0.64–3.81; *p* = 0.33) patients. Non-significant results were also observed for RFS in localized patients (HR, 0.71; 95% CI, 0.34–1.51; *p* = 0.38) and PFS in metastatic patients (HR, 0.59; 95% CI, 0.23–1.51; *p* = 0.27).

## 3. Discussion

Dystrophin expression and somatic mutations in the *DMD*, DGC-coding genes and *DYSF* were evaluated for their potential as prognostic factors in patients with localized or metastatic LMS. The findings revealed a significant association between low dystrophin expression and tumor grade. Significant associations of high dystrophin expression with poorer survival in patients with localized grade 2 tumors were observed. This study also describes the frequency of somatic mutations in *DMD* and their impact on dystrophin expression, in addition to the frequency of mutations in the DGC-coding genes in LMS.

It is well known that CNVs are the most common mutations in *DMD*, probably because *DMD* lies within a common fragile site characterized by genomic instability [18]. *DMD* deletions were identified in 16% of tumor tissues, consistent with previous studies [15,16], whereas 28% of the tumors had *DMD* amplifications. This higher frequency of amplifications agrees with the metrics from cBioportal website [19], which uses data from The Cancer Genome Atlas (TCGA) project, showing that somatic *DMD* amplifications are more frequent than deletions in sarcomas, including LMS [20]. Additionally, 89% of samples with *DMD* amplifications showed low dystrophin expression in this study. The majority of the previous studies [10,15] did not report amplifications, even though these mutations can also lead to loss of dystrophin, as described in Duchenne and Becker muscular dystrophinopathies [21].

Three mutations (deletions or amplifications) were identified in tumors with high dystrophin expression, suggesting that these mutations are likely benign. On the other hand, no mutations were identified in 54% of the samples with loss of dystrophin expression. This suggests transcriptional and translational regulation during oncogenesis or the presence of complex gene rearrangements or deep intronic *DMD* variants undetectable by MLPA and conventional exon and flanking intronic sequencing but detectable by Long-Read Sequencing [22]. This result is consistent with previously reported data on LMS [10] and several other cancers [23], where mutations were not detected in several samples with no dystrophin expression. Additionally, point mutations, mainly nonsense or frameshift mutations, also result in dystrophin loss [21]. A stop-gained *DMD* mutation (p.Tyr2141Ter) was found in a tumor with undetectable dystrophin expression. Three missense mutations (p.Lys3605Asn, p.Phe590Ile and p.Leu2734Met) were also identified. Although none of them had been previously described as pathogenic, dystrophin expression was low in two of the samples (see Table 4).

Dystrophin expression was low in 66% of the tumors, similar to previous findings [10]. The analysis of clinicopathological characteristics revealed that low dystrophin expression was more frequent in high-grade tumors. This association agrees with the findings of Wang et al. [10], who reported a higher frequency of *DMD* deletions in high-grade myogenic cancers, leading them to propose dystrophin as a biomarker of tumor aggressiveness. Additionally, 75% of the patients in our cohort with metastatic disease showed low dystrophin expression, compared to 63% of those with localized tumors, in line with a study [23] that found an association between low *DMD* expression and advanced stages in various cancers. Altogether, our findings suggest that although somatic *DMD* mutations and tumoral dystrophin expression were correlated, and most studies focused on *DMD* deletions [10,15], dystrophin expression may be a more accurate biomarker in tumor samples.

Regardless of these encouraging findings, survival analyses in patients with localized or metastatic LMS showed no significant differences according to dystrophin expression. The potential utility of dystrophin to stratify grade 2 tumors was then investigated, given the less predictable behavior of these tumors and the association observed with tumor grade. Survival analyses in patients with localized grade 2 tumors showed significantly better OS and RFS among those whose tumors expressed low levels of dystrophin. Assuming low dystrophin expression could be a biomarker of tumor aggressiveness, this significant association was unexpected. Worse OS has been reported in patients with low *DMD* expression, based on the analysis of data from 15 tumor types in the TCGA dataset, including sarcoma [23]. Analyses of *DMD* expression data from public repositories in several non-myogenic cancers also revealed that in most cases, patients whose tumors harbored *DMD* mutations showed significantly poorer OS [11]. However, in line with our findings, high dystrophin expression has been associated with shorter survival in B-cell chronic lymphocytic leukemia [24] and in low-grade glioma [25]. Note that most of these studies have analyzed *DMD* expression in RNA samples [11,23,25], although the protein can undergo post-translational regulation modulated by tissue type and/or the tumor microenvironment that may not be reflected in mRNA levels. In contrast, the variable analyzed in this study was full-length dystrophin (Dp427) assessed by IHC.

Additionally, the molecular mechanisms underlying the putative complex function of dystrophin in tumorigenesis have not yet been fully characterized. Dp427 has been shown to inhibit tumor-associated processes [10], while the ubiquitously expressed truncated Dp71 isoform has been shown to be involved in the growth of myogenic tumor cells [10,15]. Jones et al. [20] proposed that, in cancer, the ratio between full-length dystrophin and Dp71 isoforms may be altered. The impact of this presumed dysregulation of dystrophin isoforms expression may further depend on the tumor microenvironment and tumor type. Therefore, the apparently contradictory associations of low dystrophin expression with both high-grade tumors and improved survival in localized grade 2 tumors may reflect the complexity of DMD expression regulation. Further studies, including functional assays, are needed to elucidate the role of dystrophin in leiomyosarcomas.

Dystrophin further mediates its functions by interacting with proteins of the DGC. Genetic mutations in their coding genes may also contribute to tumorigenesis in LMS, as has been previously reported for *UTRN* mutations in various cancers [26], and *LAMA2* mutations in hepatocellular carcinoma [27] and olfactory neuroblastoma [28]. In this study, a stop-gained mutation in *UTRN* and a frameshift mutation in *SGCD* were identified in LMS. Utrophin expression is normally restricted to the neuromuscular and myotendinous junctions in normal adult muscle, but it relocates to the sarcolemma in dystrophin-negative fibers in Duchenne muscular dystrophy, suggesting a compensatory role [29]. The identified *UTRN* stop-gained mutation in a LMS tumor with low dystrophin expression and *DMD* amplification may disrupt this mechanism, contributing to membrane instability and signaling dysregulation, although further functional studies are required to confirm its pathogenicity. Additionally, possible deleterious missense and 3′-UTR variants were found in 12 tumors. The presence of mutations in the DGC-coding genes, *DYSF* and *DMD*, led us to analyze whether the mutational status of these genes was associated with clinicopathological characteristics and survival, but no significant associations were found. These findings do not support a significant effect of mutations in the DGC-coding genes in LMS pathogenesis. However, this is an exploratory study, and further research is required before drawing conclusions. In addition, a significant association was found between mutational status and female sex, which may be explained by the overrepresentation of uterine LMS samples, characterized by higher genetic complexity, including point mutations and chromosomal abnormalities [16,30].

To our knowledge, although modest in size, this is one of the most comprehensive studies of *DMD* molecular alterations, including both genetic variations and protein expression, in LMS patients. Also, it was the first study to evaluate mutations in the DGC-coding genes and *DYSF* as prognostic biomarkers in LMS. However, several limitations should be acknowledged. First, the exclusion of samples with poor DNA quality or multiple altered exons in *DMD* resulted in a reduced sample size in the analyses involving CNVs. However, this was the most appropriate approach due to difficulties in interpretation, as the complex genetic alterations in these samples were probably the result of high fragmentation of the FFPE material or the presence of multiple clones within the tumor. Second, the correlation between *DMD* mutations and dystrophin expression was significant, but not fully concordant. Some of the discordant cases could have been resolved by mRNA sequencing; however, mRNA was not available. Finally, this study should be regarded as exploratory, as the associations did not remain significant after Bonferroni correction for multiple comparisons. However, this method can be overly conservative in the context of genetic association studies involving correlated variables.

The proposal of *DMD* as a tumor suppressor gene [10] pointed to the potential of dystrophin as a prognostic biomarker. This study, conducted in a real-world patient cohort of LMS, supports dystrophin expression as a biomarker of tumor aggressiveness, but its role as a prognostic biomarker remains uncertain in LMS, highlighting the need for further research.

## 4. Materials and Methods

### 4.1. Study Design and Patients

This study included patients previously diagnosed with LMS and recruited at Hospital de la Santa Creu i Sant Pau (Barcelona, Spain) between 2017 and 2018. For all the patients, representative archived formalin-fixed paraffin-embedded (FFPE) primary tumor tissues were available at diagnosis. Participants’ clinical data, including age at diagnosis, biological sex, tumor site, and stage were recorded (Table 1).

### 4.2. Outcome Definitions

RFS was defined as the time from diagnosis to local or distant disease recurrence or death from any cause, whichever occurred first, for patients with localized disease. PFS was defined as the time from diagnosis to disease progression or death from any cause, whichever occurred first, for patients with metastatic disease. OS was defined as the time from diagnosis to death from any cause, for all patients.

### 4.3. Dystrophin IHC

Briefly, deparaffinized and rehydrated 4 μm thick FFPE tissue sections were heated for antigen retrieval and then incubated with the dystrophin mouse monoclonal primary antibody recognizing the rod domain of dystrophin (NCL-DYSA; Novocastra Laboratories Ltd., Newcastle Upon Tyne, UK; 1:5) and the EnVision™ FLEX/HRP, SM805 (Agilent Dako, Santa Clara, CA, USA). The antibody recognizes the 427 kDa full-length dystrophin protein (Dp427), which will be denoted as dystrophin or Dp427 throughout the text. The slides were treated with the chromogen 3,3′-diaminobenzidine tetrahydrochloride for signal detection, counterstained with haematoxylin, and mounted. Immunohistochemical stain was semi-quantitatively scored for percentage and intensity by experienced pathologists (R.O., S.B. and C.F.). The percentage of positive tumor cells was scored from 0% to 100%, and the intensity was scored as negative (0), weak (1), moderate (2) or strong (3). The dystrophin expression pattern was classified as high for intensity scores 2 to 3 and as low for intensity scores 0 to 1.

### 4.4. DNA Samples

Tissue sections were processed for DNA extraction. DNAs for genomic analyses were obtained using the GeneRead DNA FFPE Kit (Qiagen, Hilden, Germany). DNA quality was checked using the NanoDrop 2000 spectrophotometer (Thermo Fisher Scientific, Wilmington, DE, USA), and DNA concentration was measured using the Qubit dsDNA HS Assay Kits on a Qubit 4 Fluorometer (Invitrogen, Carlsbad, CA, USA).

### 4.5. Copy Number Analysis

CNVs of *DMD* were determined using the SALSA MLPA kit P034 and P035 (MRC-Holland, Amsterdam, The Netherlands) with the multiplex ligation-dependent probe amplification (MLPA) technique following the manufacturer’s instructions. The DNA amplified products were analyzed on an ABI 3100 genetic analysis system using the GeneScan™ 500 LIZ™ size standard (Applied Biosystems, Thermo Fisher Scientific, Waltham, MA, USA). The analysis of the electropherograms was performed using the Coffalyser.Net software (version 24.0, MRC-Holland, Amsterdam, The Netherlands). Three DNA samples from tonsil tissue, expected to be of normal copy number, were used as reference. The results were categorized as normal copy number, deletion or amplification.

### 4.6. DNA Library Preparation and Next-Generation Sequencing (NGS)

A custom amplicon panel (Ampliseq for Illumina; Illumina, San Diego, CA, USA) for tumor DNA samples was designed to detect mutations in selected genes encoding the main proteins of the dystrophin-associated protein complex. The sequenced genes were as follows: *DAG1*, *DMD*, *DTNA*, *DYSF*, *LAMA2*, *SGCA*, *SGCB*, *SGCD*, *SGCG*, *SNTA1*, *SNTB1*, *SSPN*, and *UTRN*. The *ACTB* gene was not well covered, so it was excluded. The panel consisted of 971 amplicons in two primer pools with an average amplicon length of 125–140 base pairs. The expected coverage of the DNA regions of interest was 93%. DNA libraries were prepared using the AmpliSeq Library PLUS and the AmpliSeq CD Indexes for Illumina (Illumina, San Diego, CA, USA) according to the manufacturer’s instructions. The quality control of the libraries was performed as follows: size was determined using the QIAxcel DNA screening gel cartridge on a QIAxcel capillary electrophoresis system (Qiagen GmbH, Hilden, Germany) and concentration using the Qubit dsDNA HS Assay Kits on a Qubit 4 Fluorometer (Invitrogen, Carlsbad, CA, USA). DNA libraries were sequenced using the MiSeq v2-300 Reagent Kit on a Miseq platform (Illumina, San Diego, CA, USA).

### 4.7. Genetic Variant Analyses

The DNA Amplicon workflow (Illumina, San Diego, CA, USA) was used for the analysis of the sequence reads (FastQ files): Burrows Wheeler Aligner was used for alignment against the human reference sequence (GRCh37/hg19), Somatic Variant Caller was used for variant calling, and Refseq was used for annotation. Variants with quality values 100 and pass filter were included in the analyses. Variants were classified according to American College of Medical Genetics and Genomics and Association for Molecular Pathology guidelines [31] REF. In silico analyses were performed using SIFT, [32], PolyPhen-2 [33] and Alamut Plus software version 1.2 (SOPHiA GENETICS SA, Lausanne, Switzerland). Only variants with putative functional effects (nonsense or frameshift mutations, possible deleterious missense or 3′-UTR variants) were included in the analysis.

### 4.8. Statistical Analyses

Categorical variables are reported as counts and percentages. Continuous variables are reported as median and interquartile range or as median with range. The Chi-square or Fisher’s exact tests were used to compare categorical variables based on sample size, along with the ordinal regression method to assess the presence of linear relationships between the variables. ORs with 95% CIs were calculated. The log-rank test was used to compare differences in OS, PFS and RFS between groups. The Kaplan–Meier method was used to estimate the survival curves and median values for each outcome. Multivariate Cox proportional hazards regression models were estimated, including primary tumor location as a covariate. HRs with 95% CIs were reported. *p*-values < 0.05 were deemed statistically significant. Multiple comparisons were controlled using the Bonferroni method, with a significance threshold set at *p* < 0.003 (19 tests). Statistical analyses were conducted using IBM SPSS Statistics (version 29.0).

## Figures and Tables

**Table 1:** Clinical and pathological characteristics of leiomyosarcoma patients.

Characteristic		n (%)
Age at diagnosis, median (range) years		77 (29–86)
Sex		
	Female	53 (69)
	Male	24 (31)
Primary tumor location		
	Extremities	31 (40)
	Uterus	25 (33)
	Retroperitoneum	21 (27)
Grade		
	1	3 (4)
	2	31 (40)
	3	42 (55)
	Unknown	1 (1)
Stage		
	Localized disease	49 (64)
	Metastatic disease	28 (36)
Death	Yes	55 (71)
Progression	Yes	23 (30)
Recurrence	Yes	36 (47)

**Table 2:** Analyses of dystrophin expression and mutational status based on variants in *DMD* and DGC-coding genes and their association with clinical, pathological and demographic variables.

	Dystrophin Expression (n = 76)				Mutational Status (n = 64)			
	High n (%)	Low n (%)	*p*-Value	OR (95% CI)	No n (%)	Yes n (%)	*p*-Value	OR (95% CI)
Stage at diagnosis								
Localized	18 (37)	30 (63)		Reference (1)	16 (40)	24 (60)		Reference (1)
Metastatic	7 (25)	21 (75)	0.26	1.79 (0.64–5)	10 (42)	14 (58)	0.9	1.07 (0.38–2.99)
Primary location								
Extremity	6 (24)	19 (76)		Reference (1)	7 (33)	14 (67)		Reference (1)
Retroperitoneal	7 (33)	14 (67)	0.26	1.33 (0.42–4.27)	7 (39)	11 (61)	0.68	1.45 (0.42–4.98)
Uterin	12 (40)	18 (60)	0.17	2.11 (0.65–6.82)	12 (48)	13 (52)	0.85	1.85 (0.56–6.13)
Tumor grade			**0.046**	2.65 (1.01–6.91)			0.20	NA (NA-NA)
Grade 1	2 (67)	1 (33)			0 (0)	1 (100)		
Grade 2	13 (42)	18 (58)			13 (52)	12 (48)		
Grade 3	10 (24)	32 (76)			12 (32)	25 (68)		
Sex								
Male	8 (35)	15 (65)		Reference (1)	12 (67)	6 (33)		Reference (1)
Female	17 (32)	36 (68)	0.8	1.13 (0.4–3.18)	14 (30)	32 (70)	**0.008**	4.57 (1.43–14.71)

Dystrophin expression: high (scores 2–3) and low (scores 0–1); Mutational status: No (no mutation) and Yes (mutation present). NA: Not Available. Statistically significant *p*-values are marked in bold.

**Table 3:** Somatic copy-number variants (CNVs) detected in the dystrophin gene (*DMD*) gene in leiomyosarcoma tumors.

Sample ID	Sex	Tumor Localization	CNV	Dystrophin Expression
18K09	Female	extremities	amplification	low
18K14	Female	uterus	deletion	low
18K16	Male	extremities	deletion	low
18K18	Female	uterus	deletion	low
18K19	Female	uterus	amplification	low
18K20	Female	uterus	amplification	low
18K21	Female	retroperitoneum	deletion	high
18K22	Male	extremities	amplification	low
18K24	Female	uterus	amplification	low
18K29	Female	retroperitoneum	amplification	low
18K30	Female	extremities	amplification	low
18K32	Female	uterus	deletion	low
18K38	Female	uterus	deletion	low
18K44	Female	retroperitoneum	deletion	low
18K50	Female	uterus	deletion	low
18K52	Male	extremities	deletion	low
18K53	Female	retroperitoneum	amplification	low
18K57	Male	retroperitoneum	amplification	high
18K61	Female	retroperitoneum	amplification	low
18K63	Female	extremities	amplification	low
18K70	Female	retroperitoneum	amplification	low
18K71	Female	retroperitoneum	amplification	low
20K20	Female	uterus	deletion	low
20K23	Female	uterus	amplification	low
20K32	Female	retroperitoneum	amplification	high
20K34	Female	extremities	amplification	low
20K37	Female	extremities	amplification	low
20K48	Female	extremities	amplification	low

ID identifier; low (scores 0–1); high (scores 2–3).

**Table 4:** Somatic mutations detected in the dystrophin–glycoprotein complex (DGC)-coding genes in leiomyosarcoma tumors.

Sample ID	Sex	Primary Tumor Localization	Gene Symbol	Nucleotide; Amino Acid	Variant Allele Fraction	Consequence (Variant)
18K1	Female	extremities	*UTRN*	c.1559G>A; p.(Arg520His)	0.53	missense
18K1	Female	extremities	*UTRN*	c.1967T>A; p.(Val656Asp)	0.49	missense
18K2	Female	retroperitoneum	*DYSF*	c.*53G>A	0.07	3 prime UTR
18k14 ^ca^	Female	uterus	*SGCD*	c.242delG; p.(Gly81GlufsTer32)	0.38	frameshift
18k19 ^ca^	Female	uterus	*UTRN*	c.571C>T; p.(Arg191Ter)	0.39	stop gained
18k21 ^cb^	Female	retroperitoneum	*UTRN*	c.5769G>C; p.(Gln1923His)	0.62	missense
18K25	Female	uterus	*DYSF*	c.*53G>A	0.47	3 prime UTR
18K36	Female	retroperitoneum	*SGCG*	c.131T>C; p.(Leu44Pro)	0.02	missense
18k36	Female	retroperitoneum	*UTRN*	c.5899C>T; p.(Arg1967Trp)	0.5	missense
18k41 ^a^	Male	extremities	*DMD*	c.6423C>A; p.(Tyr2141Ter)	0.37	stop gained
18k49 ^b^	Male	extremities	*DMD*	c.1768T>A; p.(Phe590Ile)	0.14	missense
18k55 ^a^	Female	uterus	*DMD*	c.8200C>A; p.(Leu2734Met)	0.19	missense
18K67	Female	uterus	*DYSF*	c.*53G>A	0.3	3 prime UTR
18k69	Female	extremities	*UTRN*	c.10060G>C; p.(Glu3354Gln)	0.02	missense
18k71 ^ca^	Female	retroperitoneum	*DYSF*	c.2020A>G; p.(Lys674Glu)	0.81	missense
18k71 ^ca^	Female	retroperitoneum	*UTRN*	c.8327A>G; p.(Asp2776Gly)	0.35	missense
20K16	Female	uterus	*LAMA2*	c.1316C>G; p.(Pro439Arg)	0.53	missense
20K18	Male	extremities	*SNTB1*	c.1307C>T; p.(Ala436Val)	0.47	missense
20K23 ^ca^	Female	uterus	*DYSF*	c.3678C>G; p.(Ile1226Met)	0.59	missense
20K35 ^a^	Female	uterus	*DMD*	c.10815A>C; p.(Lys3605Asn)	0.59	missense
20K41	Female	uterus	*LAMA2*	c.2992C>T; p.(Arg998Cys)	0.51	missense

*UTRN* Utrophin, *SGCD* Sarcoglycan Delta, *DYSF* Dysferlin, *DMD* Dystrophin, *SGCG* Sarcoglycan, Gamma, *LAMA2* Laminin Subunit Alpha 2, *SNTB1* Syntrophin Beta 1. Label in accordance with the assigned accession numbers: NM_007124.2 (*UTRN*), NM_000337.5 (*SGCD*), NM_001130987.1 (*DYSF*), NM_004006.2 (*DMD*), NM_000231.2 (*SGCG*), NM_000426.3 (*LAMA2*), NM_021021.3 (*SNTB1*). ^a^ Tumor sample with low dystrophin expression. ^b^ Tumor sample with high dystrophin expression. ^c^ Tumor sample with concomitant *DMD* mutations.

## Data Availability

The original contributions presented in this study are included in the article. Further inquiries can be directed to the corresponding authors.
